# Endothelial‐Derived Extracellular Vesicles During Exercise in COPD Patients

**DOI:** 10.1111/crj.70146

**Published:** 2025-12-07

**Authors:** Samuel C. Okpechi, Dorota Wyczechowska, Bennett DeBoisblanc, Jessica L. Johnson, Mohamed A. Ghonim, Natalie Bauer, Hamid A. Boulares, Matthew R. Lammi

**Affiliations:** ^1^ Stanley S. Scott Cancer Center Louisiana State University Health Sciences Center New Orleans Louisiana USA; ^2^ Department of Biochemistry and Molecular Biology Louisiana State University Health Sciences Center and School of Medicine New Orleans Louisiana USA; ^3^ Cancer Research Technology Program, Molecular Pharmacology Program Frederick National Laboratory for Cancer Research Frederick Maryland USA; ^4^ Section of Pulmonary/Critical Care and Allergy/Immunology Louisiana State University Health Sciences Center New Orleans Louisiana USA; ^5^ Comprehensive Pulmonary Hypertension Center University Medical Center New Orleans Louisiana USA; ^6^ College of Health and Pharmacy Husson University School of Pharmacy Bangor Maine USA; ^7^ The Department of Microbiology and Immunology, Faculty of Pharmacy Al‐Azhar University Cairo Egypt; ^8^ Department of Host‐Microbe Interactions St. Jude Children's Research Hospital Memphis Tennessee USA; ^9^ Department of Pharmacology and Center for Lung Biology University of South Alabama College of Medicine Mobile Alabama USA; ^10^ Division of Pulmonary and Critical Care Medicine Johns Hopkins University School of Medicine Baltimore Maryland USA

## Abstract

**Introduction:**

There are no treatments directly targeting the pulmonary vasculature in chronic obstructive pulmonary disease (COPD), and further characterization of the underlying endothelial cell (EC) abnormalities could be helpful in drug development.

**Methods:**

We investigated the influence of exercise and the prostacyclin analog iloprost on extracellular vesicles derived from ECs (eEVs) in 15 moderate–severe COPD patients who were enrolled in a randomized, placebo‐controlled crossover trial of iloprost.

**Results:**

Active smokers had a profile consistent with inflammatory‐derived EVs, while exacerbation‐prone COPD subjects had a profile consistent with apoptosis‐derived eEVs. There were no significant effects of iloprost on eEV levels. However, there was a significant increase in CD144+ and CD31+/CD144+ EVs 1 h after exercise.

**Conclusions:**

Endothelial‐derived EV profiles differed based on smoking and exacerbation history. Iloprost did not affect eEV levels, although maximal exercise induced a delayed increase in a subset of eEVs, possibly through shear stress.

## Graphical abstract

In this secondary analysis of a placebo‐controlled crossover trial enrolling COPD patients, there was no difference in exercise‐induced changes of endothelial‐derived extracellular vesicles (eEVs) between iloprost and placebo. The eEV profile was different based on smoking and exacerbation history. Compared to baseline values, eEV levels increased 1 h after maximal exercise.

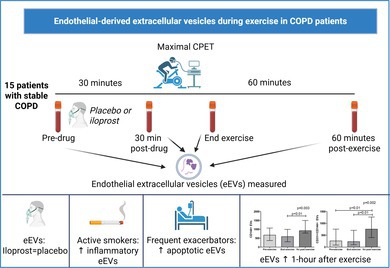

Chronic obstructive pulmonary disease (COPD) is characterized by endothelial cell (EC) abnormalities [1, 2] that play a role in disease pathogenesis. Extracellular vesicles (EVs) are submicron parent cell‐derived bilayer membranes containing biological molecules that facilitate cellular signaling [3] and have been implicated in COPD pathogenesis through inflammation/oxidative stress, airway fibrosis, and protease‐anti‐protease regulation [4]. The physiological impacts of shear stress on ECs can be investigated through the quantification of endothelial‐derived EVs (eEVs), which are elevated in stable COPD subjects [1], rise during exacerbations [1], and correlate with disease severity and IL‐6 [5]. Less is known about the impact of exercise on eEV levels in COPD. We hypothesized that eEV levels rise in COPD patients following maximal exercise due to endothelial shear stress, and that this effect would be ameliorated by the pulmonary hypertension medication iloprost, a prostacyclin analog vasodilator shown to reduce platelet aggregation [6] and improve microvascular function [7].

## 1 Methods

### 1.1 Patient Selection and Exercise Testing

This study analyzed data from 15 patients with stable moderate–severe COPD who participated in a randomized crossover trial testing the effects of 5 μg inhaled iloprost or placebo [8]. Pulmonary hypertension was not required for entry into the trial. Each subject performed a maximal cardiopulmonary exercise test (CPET) 30 min following study drug administration. Peripheral arterial blood was collected prior to study drug, 30 min after study drug (immediately prior to exercise), end‐exercise, and 1 h after exercise.

### 1.2 Isolation and Quantification of eEVs

Blood samples were collected from patients and immediately centrifuged to obtain platelet‐free plasma (PFP). The PFP was ultracentrifuged at 100,000 × *g* for 1 h at 4°C to generate the EV pellet, as previously described [9, 10]. EVs (Table 1) were selectively stained using a cocktail of antibodies targeting CD42b (BD Biosciences catalog #5552472), CD62E (#551144), CD143 (557928), CD144 (#561569), and CD31 (Thermo Fischer Scientific #46‐0319‐42). Purified human FcR binding inhibitor (#14916173) was added to the antibody‐eEV mix to prevent nonspecific binding. The reaction mix was incubated at room temperature for 15 min before samples were quantified by flow cytometry, expressed as the frequency of eEVs/100 μL of plasma.

**TABLE 1:** Endothelial cell markers used for extracellular vesicles.

Marker	Common name	Function/significance
CD62E	E‐selectin	Released predominantly in response to inflammatory signals
CD31^a^	PECAM‐1	Released predominantly in response to apoptotic signals
CD144	VE‐cadherin	Major component of the adherens junction between endothelial cells
CD143	ACE	Present mainly on pulmonary capillary endothelial cells

^a^ CD31 is also expressed on platelets and inflammatory cells.

### 1.3 Statistical Analysis

Differences between iloprost and placebo in the absolute change of eEV level from baseline across multiple time points (predrug to pre‐exercise, pre‐exercise to end‐exercise, and pre‐exercise to 1‐h postexercise) were compared using the Wilcoxon matched‐pair signed rank test. As there was no significant overall effect of iloprost on the change in eEVs, and the treatment‐by‐time interaction was not significant in a mixed effects model accounting for the crossover design (data not shown), the placebo and iloprost data were combined to test the effect of exercise on eEVs. eEVs were compared between time points using the repeated measures Friedman test with Dunn's multiple comparison.

## 2 Results

### 2.1 Patient Clinical Characteristics and eEV Levels

Fifteen moderate–severe COPD patients were included (60 ± 6 years old, 53% female, 53% active smokers, FEV_1_ 55% ± 14% predicted, peak VO_2_ 16.1 ± 4.7 mL/kg/min; Table 2). There was no correlation between log‐transformed eEV values and age, FEV_1_, or peak VO_2_. Compared to former smokers, active smokers had a lower CD31/CD62E ratio (146 [107, 262] vs. 347 [192, 2585], *p* = 0.04), indicating a relative abundance of activated/inflammatory‐derived eEVs [11]. Patients with a history of frequent exacerbations had a higher CD31/CD62E ratio than those without frequent exacerbations (347 [299, 699] vs. 146 [111, 311], *p* = 0.046), suggesting a relative increase in apoptotic‐derived eEVs [11].

**TABLE 2:** Demographic and disease characteristics of included COPD subjects.

Parameter	Mean ± standard deviation
Age (years)	60 ± 6
Pack‐years of smoking	50 ± 25
FEV_1_/FVC ratio	50 ± 13
FEV_1_ (% predicted)	55 ± 14
FVC (% predicted)	87 ± 14
RV (% predicted)	149 ± 46
TLC (% predicted)	104 ± 18
DLCO (% predicted)	57 ± 21
Peak VO_2_ ^a^ (mL/min/kg)	16.1 ± 4.7
Maximum work^a^ (Watts)	73 ± 25

Abbreviations: DLCO = diffusion capacity for carbon monoxide; FEV_1_ = forced expiratory volume in 1 s; FVC = forced vital capacity; RV = residual volume; TLC = total lung capacity; VO_2_ = oxygen consumption.

^a^ Exercise parameters displayed were on the placebo day in this crossover design.

### 2.2 Effect of Iloprost on eEV Release Before and After Exercise

The administration of iloprost was associated with increased CD143+ EVs (+17 EV/100 μL [+1, +60]) from baseline to 30 min after study drug compared to −7 EV/100 μL [−27, +4] for placebo (*p* = 0.047). There were no other treatment effects of iloprost on any of the other measured eEVs before or after exercise (all *p* > 0.05).

### 2.3 Effect of Maximal Exercise on eEV Release in COPD Subjects

**FIGURE 1:**
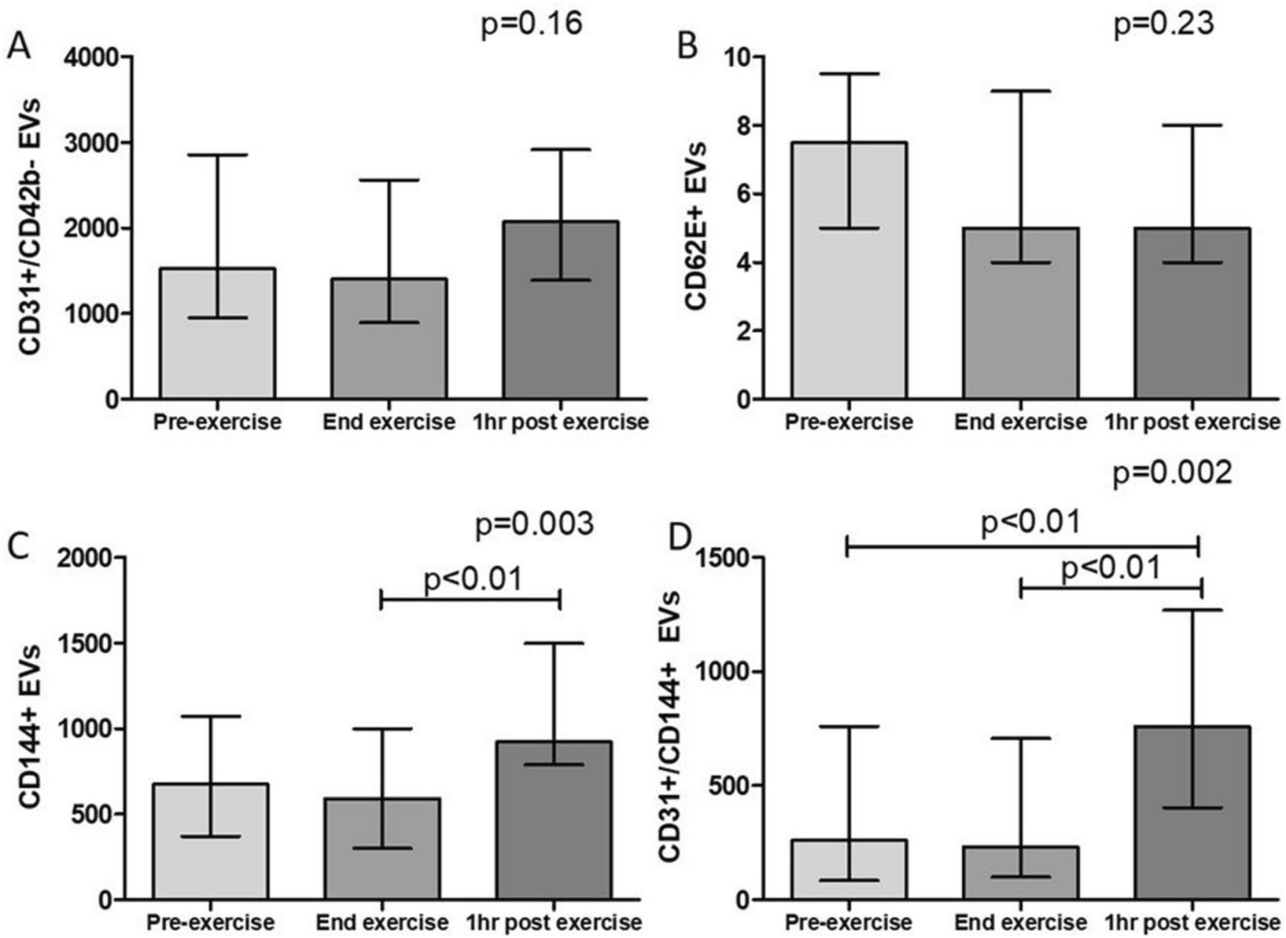
Endothelial‐derived extracellular vesicle (EV) levels in response to exercise at 3 time points: pre‐exercise, at the end of exercise, and 1 h after exercise. Values are shown as median and interquartile range for (A) CD31+/CD42b− EVs, (B) CD62E+ EVs, (C) CD144+ EVs, and (D) co‐staining CD31+/CD144+ EVs. Total sample size = 30 (15 patients on two cardiopulmonary exercise test days).

For individual patients, there was a strong correlation between the premedication values on the 2 CPET days for CD144+ eEVs (*r* = 0.77, *p* = 0.0008) and moderate correlations for CD31+ (*r* = 0.66, *p* = 0.007) and CD62E+ eEVs (*r* = 0.61, *p* = 0.02). In general, eEVs were unchanged from pre‐exercise to end‐exercise (Figure 1). However, CD144+ (*p* = 0.003) and co‐staining CD31+/CD144+ eEVs (*p* = 0.002) significantly increased 1 h after exercise. A similar, albeit nonsignificant, pattern was seen for CD31+ eEVs (*p* = 0.16).

## 3 Discussion

Although conflicting data exist, previous findings indicate that acute exercise in healthy subjects lowers eEV levels [12, 13]. Additionally, in vitro studies suggest that exercise‐induced eEVs are internalized by ECs, participating in EC repair [13]. In this secondary analysis of a randomized trial [8], we found that CD144+ and CD31/144+ eEVs significantly increased 1 h after exercise termination, consistent with evidence that eEVs increase under experimental shear stress in moderate–severe COPD subjects [14]. This delayed increase in eEVs may relate to the temporal kinetics of shear stress and endothelial activation [15], blood flow redistribution during the recovery phase altering eEV clearance dynamics [16], and/or the effects of tissue hypoxia and reoxygenation [17]. Our data conflict with a prior report of eEV levels in exercising COPD subjects, which found a decrease in EVs 1 h after exercise termination [18]. These discrepant findings may be due to cohort differences, as our subjects were younger and more likely to be female and/or active smokers, all of which influence eEV levels. Contrary to our hypothesis, inhaled iloprost administered prior to exercise did not have any significant effects on eEV levels after adjusting for multiple comparisons. Although speculative, it is possible that the iloprost dose administered was inadequate or that the interval between dosing and exercise was too long, given the short half‐life of iloprost.

There was a moderate to strong intra‐patient correlation between eEVs measured on Day 1 and Day 2 of this trial, demonstrating relative stability of these markers over a short period (median time between study visits of 3 days). Our final major finding is that the origin of eEV release differed based on COPD characteristics. Active smokers tended to have more eEVs associated with EC activation/inflammation [11]. Those with a COPD exacerbation history had a higher CD31/CD62E ratio, indicating that the eEVs were released due to EC apoptosis [11]. This is consistent with prior findings that eEVs were higher in frequent exacerbators [1]; whether eEVs can be a clinically relevant biomarker of the frequent exacerbator phenotype deserves further study.

In conclusion, our study found that eEV subtypes significantly increased 1 h after exercise termination and that this effect was not attenuated by iloprost inhalation. Future studies may focus on the contents and physiological relevance of exercise‐induced eEVs in COPD subjects, as well as on other interventions to lower EC shear stress and eEV release.

## Author Contributions

Conceptualization and study design: Matthew R. Lammi, Natalie Bauer, Hamid A. Boulares, Jessica L. Johnson, and Bennett DeBoisblanc. Data collection: Samuel C. Okpechi, Dorota Wyczechowska, Mohamed A. Ghonim, Natalie Bauer, and Hamid A. Boulares. Data analysis and interpretation: Matthew R. Lammi, Hamid A. Boulares, Samuel C. Okpechi, and Mohamed A. Ghonim. Manuscript drafting: Matthew R. Lammi and Samuel C. Okpechi. Manuscript review, revision and approval: All authors reviewed and approved the final manuscript.

## Funding

This study was supported in part by the Louisiana Clinical and Translational Sciences Center and the National Institute of General Medical Sciences grants U54‐GM‐104940 and 1‐P306‐M‐206932‐01A1 (both to MRL). The funding source had no influence on the design or conduct of this study.

## Disclosure

The authors have nothing to report.

## Ethics Statement

This study was approved by the Institutional Review Board of LSUHSC (#8163, 5/27/2013), and all participants gave written informed consent.

## Conflicts of Interest

None of the authors have relevant conflicts of interest to report.

## Data Availability

The data that support the findings of this study are available from the corresponding author upon reasonable request.
